# Seasonal Dynamics of Marine Microbial Community in the South Sea of Korea

**DOI:** 10.1371/journal.pone.0131633

**Published:** 2015-06-29

**Authors:** Sung-Suk Suh, Mirye Park, Jinik Hwang, Eui-Joon Kil, Seung Won Jung, Sukchan Lee, Taek-Kyun Lee

**Affiliations:** 1 South Sea Environment Research Department, Korea Institute of Ocean Science and Technology, Geoje, 656–830, Republic of Korea; 2 Korea University of Science and Technology, Daejeon, 305–350, Republic of Korea; 3 Department of Genetic Engineering Sungkyunkwan University, Suwon, 440–746, Republic of Korea; University of New South Wales, AUSTRALIA

## Abstract

High-resolution 16S rRNA tag pyrosequencing was used to obtain seasonal snapshots of the bacterial diversity and community structure at two locations in Gosung Bay (South Sea, Korea) over a one year period. Seasonal sampling from the water column at each site revealed highly diverse bacterial communities containing up to 900 estimated Operational Taxonomic Units (OTUs). The *Alphaproteobacteria* and *Gammaproteobacteria* were the most abundant groups, and the most frequently recorded OTUs were members of *Pelagibacter* and *Glaciecola*. In particular, it was observed that *Arcobacter*, a genus of the *Epsilonproteobacteria*, dominated during summer. In addition, *Psedoalteromonadaceae*, *Vibrionaceae* and SAR11-1 were predominant members of the OTUs found in all sampling seasons. Environmental factors significantly influenced the bacterial community structure among season, with the phosphate and nitrate concentrations contributing strongly to the spatial distribution of the *Alphaproteobacteria*; the *Gammaproteobacteria*, *Flavobacteria*, and *Actinobacteria* all showed marked negative correlations with all measured nutrients, particularly silicon dioxide and chlorophyll-a. The results suggest that seasonal changes in environmental variables contribute to the dynamic structure of the bacterial community in the study area.

## Introduction

Knowledge of the distribution and abundance of bacteria and the environmental factors that control them is crucial for understanding and predicting ecosystem function and the responses to of future changes of the ecosystem [1, 2]. A basic tenet of community ecology is that major spatial and temporal patterns in the distribution and abundance of bacterial taxa are structured and influenced by both regional (geography) and environmental factors (e.g., abiotic, biotic, nutrient or other), linking ecosystem function to biodiversity [3, 4]. Most studies of the relationship of biodiversity to ecosystem function have concerned animals and plants (macroorganisms), rather than microorganisms including bacteria, but it becoming increasingly evident that bacteria are dominant taxa among marine species, in terms of both relative abundance and their contribution to biological processes in marine ecosystems [5, 6]. However, the extent to which the interactions among environmental factors govern ecosystem and biogeochemical processes remains difficult to assess in the absence of information about microbial biodiversity patterns at equivalent resolution to that of macroorganism biodiversity.

Recent advances in next-generation sequencing techniques have enabled large-scale exploration of the taxonomic diversity and geographic distribution of marine bacteria [7, 8]. High-throughput pyrosequencing techniques for phylogenetically informative marker genes, including the ribosomal RNA (rRNA) genes, have recently provided evidence that marine bacteria may indeed exhibit spatial patterns akin to those of larger organisms, in terms of distribution and abundance, but their temporal patterns remain relatively unexplored in the ocean water column [9–12]; the finding obtained thus far have led to comprehensive descriptions of natural microbial assemblages [13, 14]. These pyrosequencing techniques have provided evidence of broad-scale patterns in the distribution and abundance of dominant taxa among the prokaryotic plankton. For example, bacterial groups including the SAR11 cluster are now known to be abundant in most ocean ecosystems, but *Cyanobacteria* and certain *Roseobacter* occur only in certain habitats [15, 16]. In addition, investigations of the temporal trends of various bacterial groups over multiple years have indicated that certain groups tend to be more common during particular seasons, showing statistically robust and predictable patterns in microbial communities [17, 18].

The diversity and composition of the microbial community at a given location are structured and influenced by both regional (geography) and local processes including species interactions, and environmental factors. Changes in such features of a community can repeat seasonally in response to seasonal variations in environmental parameters, including temperature, salinity, dissolved oxygen and chlorophyll-a. Numerous studies investigating the effect of environmental factors on microbial communities have focused primarily on the relative importance of temperature and nutrient concentrations [19, 20]. These are obvious candidates because of the strong effect of temperature on biological processes [21] and the fact that nutrient availability can drive niche structure through resource partitioning [22]. A finding of particular importance to the present study is the recent demonstration that marine bacteria follow a latitudinal diversity gradient, with maximum potential richness being primarily driven by temperature, but with many other factors modulating an intricate network of richness at any particular temperature [23]. Given the dominance of bacteria in communities and ecosystem processes, predicting the ecosystem response to environmental change requires assessment of whether bacterial populations exhibit diversity patterns. Several studies have demonstrated that it is possible to predict the taxonomic composition of bacterial communities from environmental parameters [24, 25]. For example, there are annual cyclical patterns for multiple microbial taxa in marine ecosystems that are predictable from environmental conditions [17, 24]. Such studies more directly and convincingly enable assessment of the extent to which bacterial communities may be deterministic.

In this study, we (i) assessed whether the distribution and abundance of marine bacterial taxa exhibited marked temporal patterns over seasonal periods; (ii) investigated the relationship of any pattern to abiotic and biotic environmental factors; and (iii) determined which environmental factors might have the greatest influence on diversity. The microbial consortia of Gosung Bay (South Sea, Korea) have rarely been documented. We undertook seasonal sampling of the microbial community at this location, and measured a suite of oceanographic environmental variables including temperature, salinity, dissolved oxygen, chlorophyll-a, and nutrients. The findings contribute to our knowledge of patterns of marine microbial biogeography and provide a baseline for further studies.

## Materials and Methods

### Ethics statement

No specific permits were required for the described field studies. The location is not privately-owned or protected in any way, and the field studies did not involve endangered or protected species.

### Measurement of environmental parameters

The water temperature, pH, salinity and dissolved oxygen (DO) concentration in each sub-sample were measured using a portable meter (556 MPS; YSI, USA). A 100 mL aliquot of the sub-sample was filtered through a glass fibre filter (GF/F; Whatman, USA), stored in an acid-cleaned polyethylene bottle in a deep freezer at -80°C and then the concentrations of inorganic nutrients in the sample were determined. The inorganic nutrients that were determined were dissolved inorganic nitrogen (NO_2_^−^, NO_3_^−^ and NH_4_^+^), phosphate (PO_4_^3-^) and silicate (SiO_2_). The nutrient concentrations in each sample were analysed using an automatic nutrient analyser (Lachat Quickchem; Lachat Instruments, Milwaukee, USA). To analyse chlorophyll-*a* concentrations, a 250-mL sample was filtered through a GF/F filter under low vacuum pressure. The filter was then soaked in 15 mL of cold 90% acetone-distilled water (v/v), sonicated to break cell walls, and incubated for 24 h in the dark at 4°C. Finally, the chlorophyll *a* concentration was estimated in accordance with the equation of Humphrey and Jeffrey [26].

### Sample Collection

Surface seawater samples were taken at a random depth between 0 and 5 m at two sites in Gosung Bay (N34°85'38.7''E128°23'54.9'', N34°84'02.2''E128°27'55.1'') in March, June, September and December 2013, representing winter, spring, summer and autumn, respectively. A total of ~30 L of ambient seawater was collected per sampling site per season in sterile plastic bottles, and members of the microbial community by filtering the seawater through a polycarbonate membrane filter (0.22 μm; Millipore, Billerica, MA, USA). All samples were stored at -80°C until further analysis.

### DNA extraction, PCR amplification and pyrosequencing

The filter membranes supporting microbial cells were cut into pieces before DNA extraction. Total DNA was extracted using the PowerSoil DNA Isolation Kit (MoBio, Solana Beach, CA, USA), in accordance with the manufacturer’s instructions. The total extracted DNA was used for polymerase chain reaction (PCR) amplification using primers targeting the V1–V3 region of the 16S rRNA gene. For the bacterial amplification, the barcoded primers 9F (5'-CCTATCCCCTGTGTGCCTTGGCAGTC-TCAG-AC-AGAGTTTGATCMTGGCTCAG-3'; the underlined sequence indicates the target region primer; 'CCTATCCCCTGTGTGCCTTGGCAGTC' indicates the adaptor sequence; 'TCAG' is the key sequence; 'AC' is the linker sequence) and 541R (5'-CCATCTCATCCCTGCGTGTCCGAC-TCAG-X-AC-ATTACCGCGGCTGCTGG-3'; 'X' indicates the unique barcode for each subject; 'TCAG' indicates the key sequence; 'CCATCTCATCCCTGCGTGTCCGAC' is the dose adaptor sequence; 'AC' is the dose linker sequence; 'ATTACCGCGGCTGCTGG' is the dose primer sequence) were used. Amplification was performed under the following conditions: initial denaturation at 95°C for 5 min; 30 cycles of denaturation at 95°C for 30 s, primer annealing at 55°C for 30 s and extension at 72°C for 30 s; and final extension at 72°C for 5 min. The PCR products were confirmed using electrophoresis on 2% agarose gels; bands were visualised using a Gel Doc system (BioRad, Hercules, CA, USA). The PCR products were extracted from the agarose gels using the QIAquick PCR Purification Kit (QIAGEN, Cat. # 28106). Equal amounts of purified product were pooled, and short fragments (non-target products) were removed using the Ampure bead kit (Agencourt Bioscience, Beverly, MA, USA). The product size and quality were assessed on a Bioanalyser 2100 (Agilent, Palo Alto, CA, USA) using a DNA 7500 chip. Mixed amplicons were subjected to emulsion PCR and then deposited on picotitere plates (Agilent). Sequencing was performed by Chunlab Inc. (Seoul, Korea) using the GS Junior Sequencing system (Roche Branford, CT, USA), in accordance with the manufacturer’s instructions.

### Pyrosequencing Data Analysis

The basic analysis was conducted as described previously [27]. Sequence handling and analysis were carried out following the Mothur curation pipeline v1.0c. Briefly, Fasta, quality and flow files were extracted from Roche files from each pool and flowgrams were trimmed and denoised (minflows = 360, maxflows = 720, pdiffs = 0, bdiffs = 0). Fasta files were processed by identifying perfect matches to primer and barcode sequences in the reads or the reverse complement sequences, trimming these sequences, and sequences meeting the 300 nucleotide minimum length requirement were output (pdiffs = 0, bdiffs = 0, maxhomop = 8, minlength = 300, flip = T). The number of unique sequences was also determined at this and subsequent steps in the analysis. After concatenating the read output from the two pools, the sequences or their reverse-complements were aligned to the SILVA database [28]. Sequences not aligning within the optimised alignment region were removed from the analysis with the screening function (optimise = start-end, minlength = 300, criteria = 97). Potential chimera sequences were detected using the Bellerophon method, which compares the BLASTN search results between the forward-half and reverse-half sequences. After generating distance matrices from aligned sequences and clustering OTUs using a distance of 0.03, taxonomic assignments were made using the RDP classifier v2.4 trained on dataset 7 with a confidence threshold of 0.7 at genus level and 0.97 at the domain level [29]. The overall phylogenetic distance among communities was estimated using Fast UniFrac [30] and visualised using principal coordinate analysis (PCoA) [30, 31]. To compare OTUs among samples, shared OTUs were identified using XOR analysis (CL community program; Chunlab Inc.) [32, 33].

## Results

### Diversity and composition of seawater-associated bacterial communities

**Fig 1:**
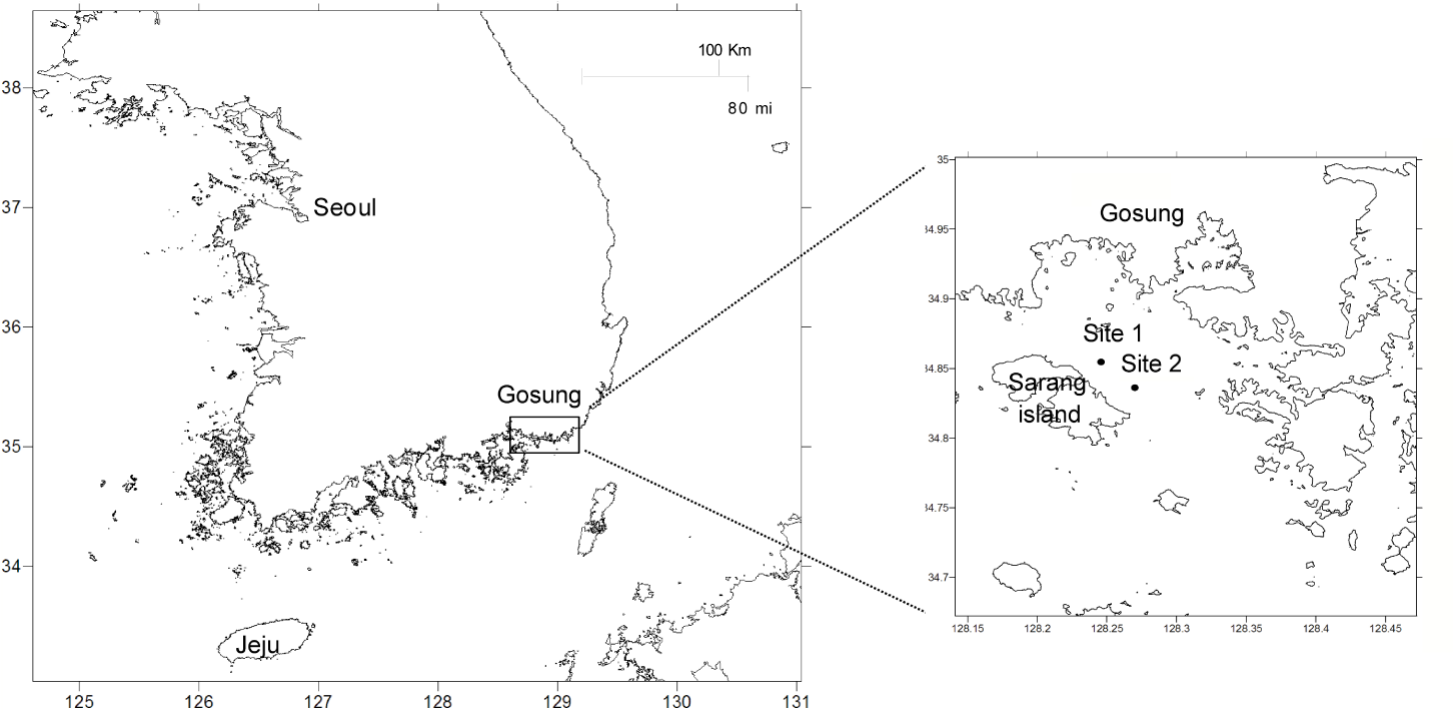
Maps showing the sampling locations in the South Sea of Korea.

To determine the composition and distribution of bacteria in the water column, seasonal samples were collected at two sites in Gosung Bay (Fig 1); environmental factors including temperature, dissolved oxygen (DO), chlorophyll-a and various nutrients differed significantly among the seasons (one-way ANOVA; *P* < 0.05) (Table 1). Pyrosequencing was performed on the mixed PCR amplicons generated from the pooled DNA from each site. A total of 53,592 reads (average number of reads per sample, 6699; average read length, 372 bp) were recovered following quality control filtering, and clustered into 15,138 OTUs (97% of the qualified reads; Table 2). The numbers of OTUs in winter, spring, summer and autumn in Gosung Bay were 1408, 1989, 1536 and 1235 OTUs, respectively. On the basis of the nonparametric Chao1 index, the richness of the entire bacterial community was highest during spring, while the bacterial diversity appeared to be similar between the sampling sites and among seasons, based on the Shannon diversity index; the exception was the spring water column, which showed much more bacterial diversity than in the other seasons.

**Table 1:** Environmental parameters at the sampling locations.

	Temp (°C)		Sal (ppt)		DO (mg/liter)		pH		Chl a (μg/liter)		NH_4_ (mg/liter)		SiO_2_ (mg/liter)		NO_2_ (mg/liter)		NO_3_ (mg/liter)		PO_4_ (mg/liter)	
	Site 1	Site 2	Site 1	Site 2	Site 1	Site 2	Site 1	Site 2	Site 1	Site 2	Site 1	Site 2	Site 1	Site 2	Site 1	Site 2	Site 1	Site 2	Site 1	Site 2
Winter	11.8 ±.0.01	12.1 ±.0.01	35.46 ±.0.01	35.38 ±.0.01	8.54 ±.0.01	8.34 ±.0.01	8.18 ±.0.01	8.15 ±.0.02	1.52 ±.0.01	1.79 ±.0.02	1.54 ±.0.05	1.50 ±.0.08	4.68 ±.0.12	4.64 ±.0.21	0.10 ±.0.01	0.097 ±.0.03	0.301 ±.0.02	0.246 ±.0.01	0.20 ±.0.01	0.24 ±.0.01
Spring	20.2 ±.0.01	20.8 ±.0.01	34.81 ±.0.02	34.76 ±.0.01	5.78 ±.0.01	5.52 ±.0.02	8.13 ±.0.02	8.11 ±.0.02	2.65 ±.0.05	2.60 ±.0.03	2.62 ±.0.17	2.19 ±.0.07	9.48 ±.0.61	9.07 ±.0.31	0.288 ±.0.02	0.188 ±.0.01	1.223 ±.0.08	1.136 ±.0.11	0.32 ±.0.02	0.29 ±.0.01
Summer	22.5 ±.0.01	22.8 ±.0.01	33.61 ±.0.02	33.23 ±.0.01	6.04 ±.0.01	6.13 ±.0.01	8.10 ±.0.01	8.12 ±.0.01	4.97 ±.0.06	4.07 ±.0.03	1.29 ±.0.01	1.60 ±.0.06	10.17 ±.0.11	10.84 ±.0.31	0.115 ±.0.01	0.141 ±.0.01	0.737 ±.0.07	0.727 ±.0.10	0.21 ±.0.01	0.21 ±.0.01
Autumn	14.6 ±.0.01	14.3 ±.0.01	34.45 ±.0.01	34.62 ±.0.01	7.65 ±.0.02	7.52 ±.0.01	8.07 ±.0.01	8.03 ±.0.01	0.71 ±.0.04	1.12 ±.0.03	0.21 ±.0.05	0.26 ±.0.03	9.41 ±.0.21	8.32 ±.0.18	0.612 ±.0.02	0.723 ±.0.01	6.550 ±.0.11	7.473 ±.0.08	0.65 ±.0.12	0.48 ±.0.05

Temperature (Temp), salinity (Sal), dissolved oxygen (DO), pH and chlorophyll-a (Chl-a) content were measured with a YSI 6600 Sonde; nitrate, ammonium and phosphate were measured with a nutrient analyser. The data are presented as mean ± standard deviation of three measurements.

**Table 2:** Numbers of sequences and OTUs (97%) and diversity estimates of bacteria.

Index	Winter		Spring		Summer		Autumn	
	Site 1	Site 2	Site 1	Site 2	Site 1	Site 2	Site 1	Site 2
No. of Seq	6286	5156	7031	11253	4662	9266	4944	4994
OTUs	646	762	996	993	652	884	622	613
Ace	1420.36	1731.48	2918.21	2128.71	1808.86	1979.41	1343.62	1312.36
Chao1	1087.27	1247.86	1705.00	2054.66	1228.18	1486.67	1117.23	987.67
Shannon	4.648	5.154	5.255	4.858	4.726	4.800	4.686	4.750

Number of sequences and OTUs (97%) and diversity (TDC-TBC) estimates bacteria

**Fig 2:**
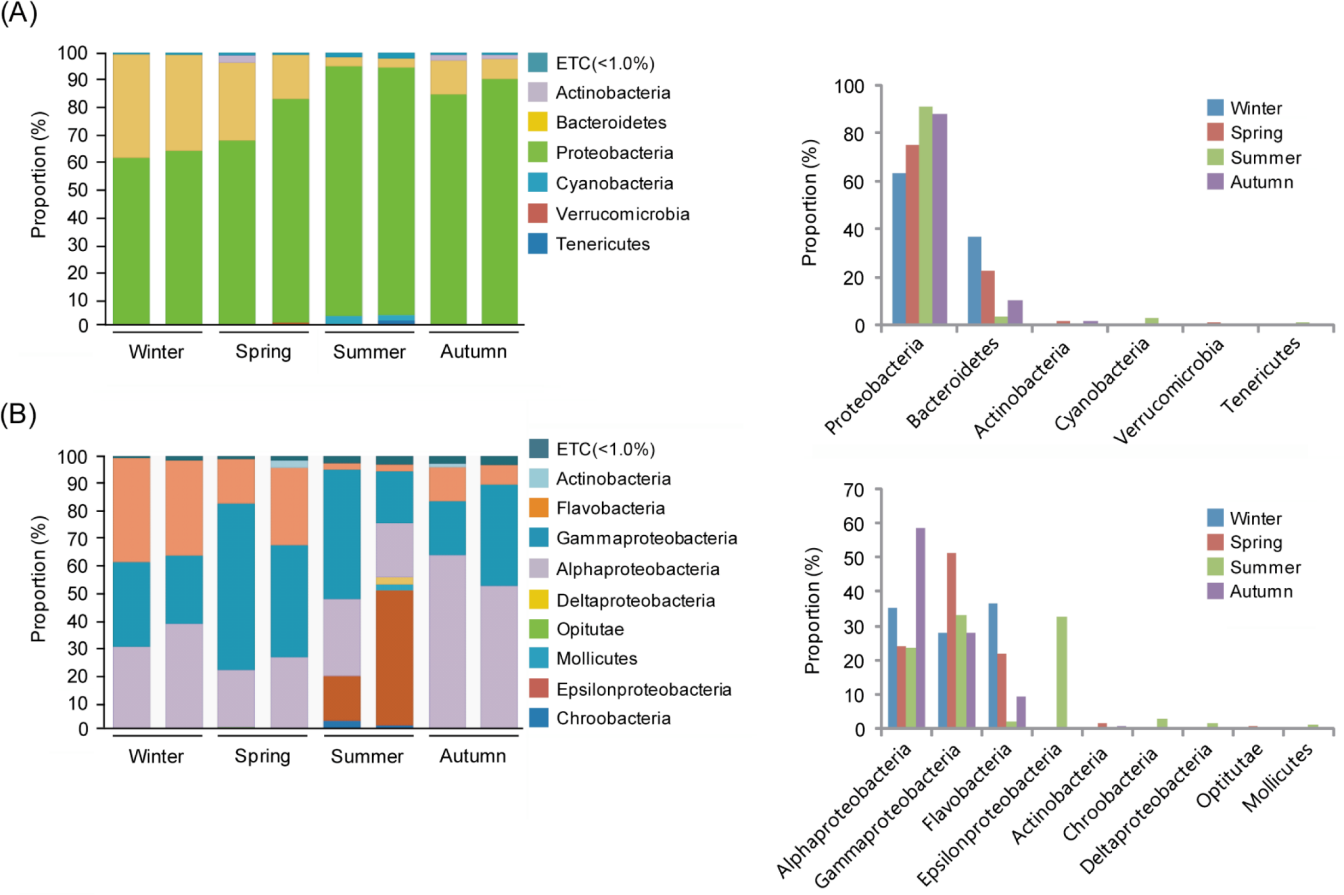
Taxonomic classification of bacterial reads retrieved from pooled DNA amplicons from different seasonal water masses into phylum (A) and class (B) levels using the RDP classifier.

The 53,592 quality-controlled reads were assigned to 13 described bacterial phyla, with six ubiquitous phyla making up an average of 97.8% of the reads (*Proteobacteria*, *Bacteroidetes*, *Cyanobacteria*, *Actinobacteria*, *Verrucomicrobia and Tenericutes*) (Fig 2A). The proportions of these five taxa in the water samples from the two sites varied among the seasons; however, *Proteobacteria* were most abundant, comprising 63%–95% of the bacterial reads in all seawater samples. The proportion of this taxon gradually increased from winter to summer, but decreased a little during autumn. The second most abundant group was *Bacteroidetes*, which showed the opposite distributional pattern to *Proteobacteria*; the proportion of *Bacteroides* decreased significantly from spring to summer, and increased during autumn. *Cyanobacteria* were only detected during summer, while *Verrucomicrobia* and *Tenericutes* were only detected during spring and summer, respectively.

Further classification to the class level indicated that bacterial communities in the water column varied considerably among the seasons (Fig 2B). For instance, winter and spring water masses were commonly dominated by the three classes, *Alphaproteobacteria*, *Gammaproteobacteria*, and *Flavobacteria*, with the majority of reads belonging to the phyla *Proteobacteria* and *Bacteroidetes*, respectively, while in summer, the seawater contained varying proportions of *Alphaproteobacteria and Gammaproteobacteria*. In addition, *Epsilonproteobacteria* and *Chroobacteria* in the phyla *Proteobacteria* and *Cyanobacteria*, respectively, were only observed in summer, whereas *Alphaproteobacteria* and *Gammaproteobacteria* were heavily represented in all seasons, and were particularly dominant in autumn and spring, respectively. The proportion of *Flavobacteria* decreased markedly in summer and autumn compared with that in winter and spring, and *Actinobacteria* were only present during autumn and spring, respectively, but even then their proportions in the samples were negligible. In addition, *Deltaproteobacteria* and *Mollicutes* were only detected during summer.

**Fig 3:**
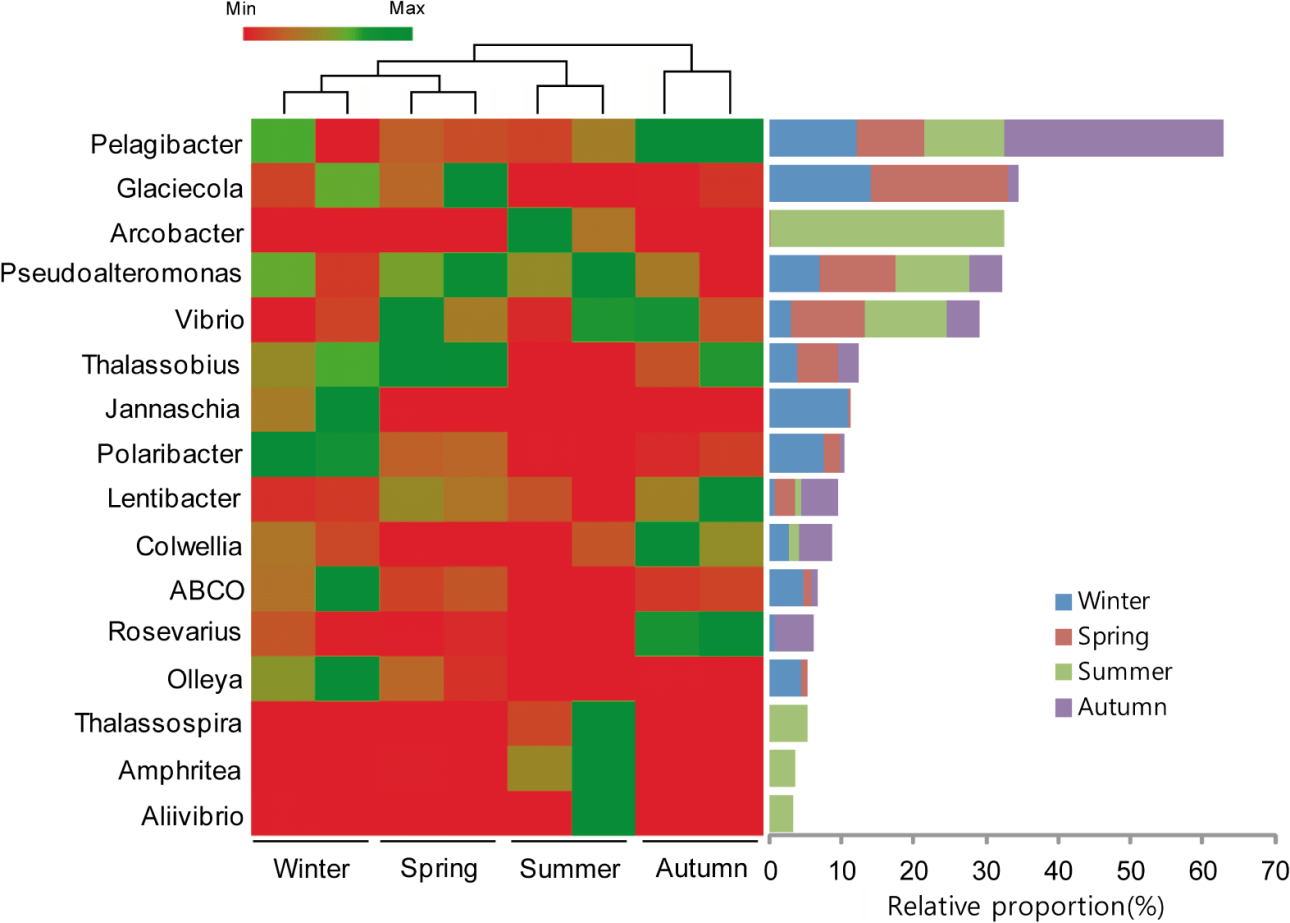
Heat map showing the relative abundances and distribution of representative 16S rRNA gene tag sequences classified at the genus level. Display with minimum ratio (5%) of the normalised abundance, using an RDP confidence level of 0.7. The normalised data were centred by the mean and clustered using the complete linkage method and a metric of correlation (uncentred). The colour code indicates differences in the relative abundance from the mean, ranging from red (negative) through black (the mean) to green (positive).

Heat map analysis of the bacterial communities at the genera level revealed distinct diversity from one season to the next for all bacterial taxa and heterogeneous temporal distributions. For example, *Pelagibacter*, the most abundant genus, was detected in all samples, and dominated more during autumn than in the other seasons (Fig 3). In addition, more sequences associated with *Prochlorococcus* were detected during winter and spring than in the other seasons, while *Pseudoalteromonas* and *Vibrio* were dominant during spring and summer. *Arcobacter*, *Thalassospira*, *Amphritea*, and *Aliivibrio* were mainly present during summer, whereas *Jannaschia* and *Polaribacter* were mainly present during winter

### Within-subject OTU overlap among the seasons

**Fig 4:**
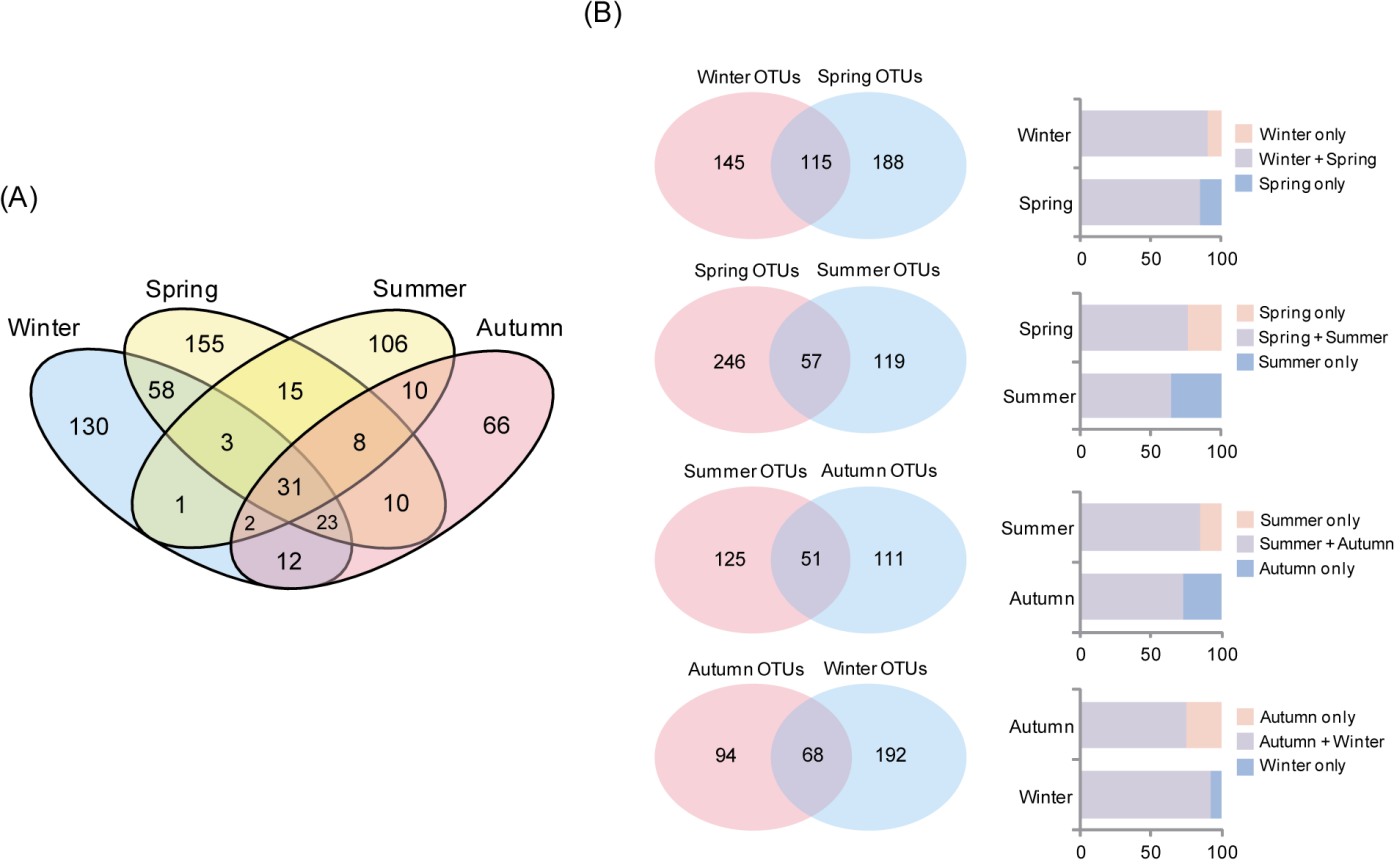
The distribution of OTUs among the seasonal bacterial communities in Gosung Bay. Venn diagrams demonstrating 97% OTU cluster overlap within all seasonal samples (A) or between two different seasons (B). Numbers correspond to unique OTU clusters within a subset. To highlight shared OTUs, singleton clusters were removed before analysis.

We further investigated the patterns of relative abundance and diversity of the microbial communities among the seasons. The distribution of OTUs in the seawater samples was investigated by combining all tag sequences and assessing their occurrence among different samples. The overlap of OTU clusters among sampling seasons (winter, spring, summer and autumn) was calculated with singleton sequences removed. The resulting Venn diagrams showed that winter and spring had the largest OTU overlap within an individual site. Both sampling sites shared a large number of OTUs in summer, while the winter and summer sets of OTUs typically had the smallest overlap (Fig 4A). In addition, all seasonal water samples shared 31 OTUs, showing that their abundance varied among the seasons. With regard to seasonal differences in bacterial diversity, the number of OTUs (n = 188) present in spring and absent in otherwise winter was higher than the number of OTUs in winter (n = 145) (Fig 4B), with bacterial abundance in spring being higher than in winter. The two seasons shared 115 OTUs in common, 110 of which were distributed in the *Flavobacteriaceae* (n = 41), *Rhodobacteriaceae* (n = 33), *Alteromonadaceae* (n = 10), *Pseudoalteromonadaceae* (n = 11), *SAR11* (n = 10), *Colwelliacea* (n = 3) and *Vibrionaceae* (n = 2), with the remaining taxa identified as belonging to bacterial groups including *Colwelliacea* and *Vibrionaceae* (Table 3). The number of OTUs present in summer and absent in spring (n = 119) was less than the number of OTUs in winter (n = 246) (Fig 4B). The summer and spring water column samples shared 57 OTUs in common, 56 of which were distributed in the *Vibrionaceae* (n = 23), *Rhodobacteraceae* (n = 9), *Pseudoalteromonadaceae* (n = 8) and *SAR11* (n = 8) taxa, with remainder identified as belonging to bacterial groups including the *Flavobacteriaceae*, *Alteromonadaceae*, and *Colwelliacea*. In addition, the OTUs common to summer and autumn water column samples (n = 51) were distributed primarily in *SAR11-1* (n = 22), *Pseudoalteromonadaceae* (n = 18), and *Vibrionaceae* (n = 16), while the OTUs common to both the autumn and the winter water columns (n = 68) belonged to the *Flavobacteriaceae* (n = 22), *Colwelliacea* (n = 19), *Pseudoalteromonadaceae* (n = 15) and *SAR11* (n = 13); their proportions in winter were higher than in autumn.

**Table 3:** The bacterial distribution of OTUS in the seasonal bacterial communities.

	*Flavobacteriaceae*	*Rhodobacteraceae*	*Alteromonadaceae*	*Colwelliacea*	*Pseudoalteromonadaceae*	*Vibrionaceae*	SAR11-1	*Campylobacteraceae*
Winter	79	26	7	2	1	2	N/A	N/A
Winter & Spring	41	33	10	3	11	2	10	N/A
Spring	52	16	50	3	10	13	N/A	N/A
Spring & Summer	5	9	2	1	8	23	8	N/A
Summer	6	11	N/A	N/A	10	9	N/A	22
Summer& Autumn	8	9	N/A	2	18	16	22	N/A
Autumn	4	19	N/A	12	2	1	2	N/A
Autumn & Winter	22	14	1	19	15	16	13	N/A

The eight most abundant bacterial classes (>1.0%) determined using an RDP confidence threshold of 0.7 were chosen.

### Statistical comparison of the microbial community structure

**Fig 5:**
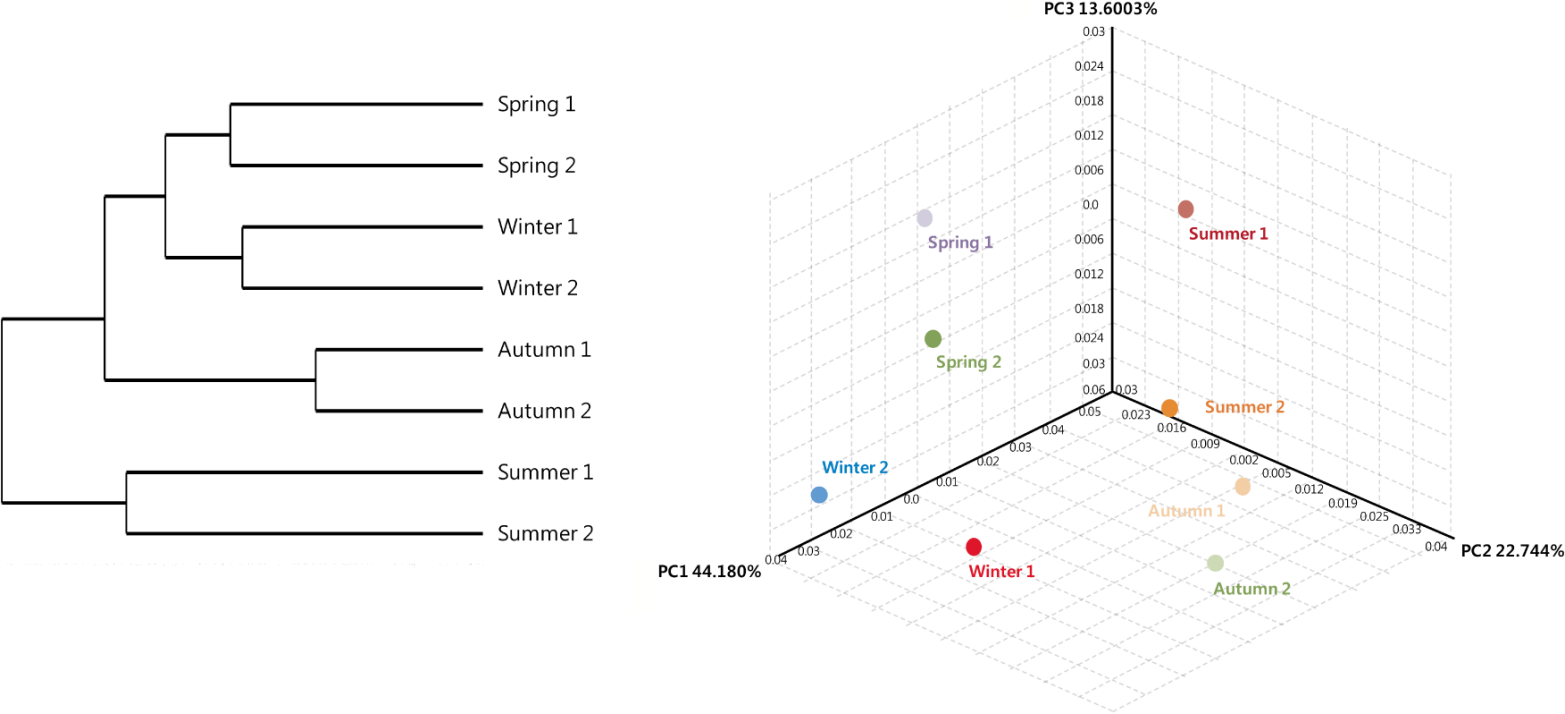
UniFrac distance-based Jackknife clustering of bacterial communities associated with different seasonal water masses from different sampling locations. Unifrac PCoA images were captured from 3D UniFrac PCoA to illustrate differences in the microbiota among the different samples. The following UniFrac PCoA analyses were based on the OTU data, with only the first three principal coordinates (PCs) shown: unweighted UniFrac with PC1 = 44.180, PC2 = 21.744, and PC3 = 13.6003% (*p* = 0.001).

**Fig 6:**
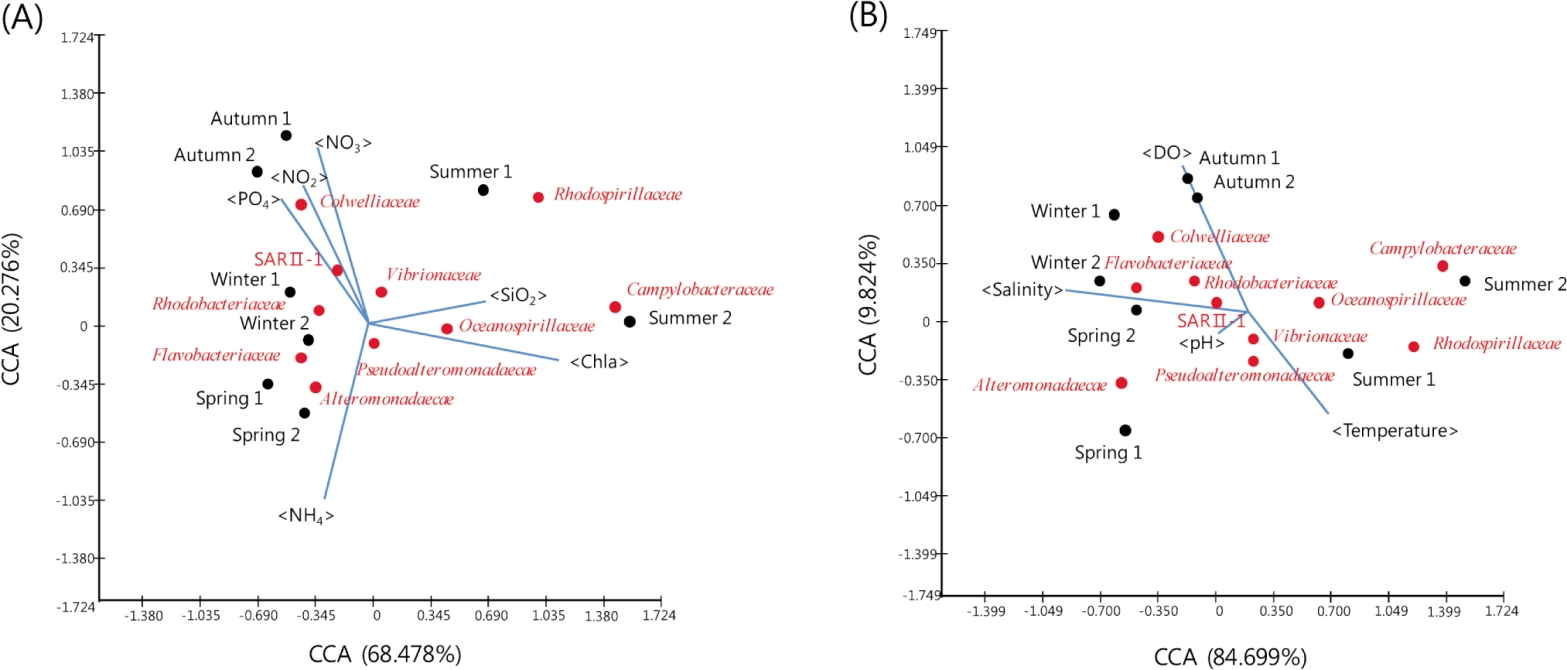
Canonical correspondence analysis (CCA) ordination diagram of bacterial communities associated with environmental variables. Triplot showing the relationship between environmental parameters (nitrate, ammonium, phosphate, silicon dioxide and chlorophyll a), sampling seasons, and bacterial compositions (A), or between environmental parameters (temperature, dissolved oxygen, salinity and pH) (B). Correlations between environmental variables and the first two CCA axes are represented by the lengths and angles of the arrows. Environmental variables are indicated as blue lines.

The constitution of 16S rRNA gene sequences among the seasonal samples was assessed using the weighted UniFrac clustering method. The statistical analysis showed that the bacterial communities in spring and winter water column samples clustered together. Interestingly, the microbial community associated with summer seawater was significantly different from the communities in other seasons, as indicated by the PCoA based on the unweighted UniFrac distance (Fig 5). Canonical correspondence analysis (CCA) results for several microbial assemblages in relation to several environmental factors are shown in Fig 6. In this figure the correlations between specific environmental factors and microbial groups are represented by the angle of the arrows between them. The data indicate that the temporal distribution of several major microbial assemblages was mainly influenced by the nutrient conditions and several other environmental factors. Phosphate and nitrate contributed substantially to the spatial distribution of the *Alphaproteobacteria*, while silicon dioxide was correlated with the occurrence of *Epsilonproteobacteria* and *Chroobacteria* (Fig 6A). In contrast, the occurrences of *Gammaproteobacteria*, *Flavobacteria*, and *Actinobacteria* were negatively correlated with all of the nutrients noted above, particularly silicon dioxide and chlorophyll-a. With regard to correlations with environmental factors (Fig 6B), salinity showed a marked correlation with the spatial distribution of *Flavobacteria* and *Actinobacteria*, the *Alphaproteobacteria* were positively correlated with dissolved oxygen (DO), and the abundance of *Epsilonproteobacteria* and *Chroobacteria* negatively correlated with the levels of all environmental factors, particularly salinity.

## Discussion

Marine bacteria play important roles in energy and matter fluxes in the sea, so an understanding of their distribution and seasonal diversity is essential. Many studies have investigated microbial distribution and diversity with regard to the particular environmental and geographical conditions in various microbial habitats [34, 35]. In this study, the bacterial communities associated with seawater collected on a seasonal basis from Gosung Bay (South Korea) were investigated over a one-year period. The absolute values and the seasonal and episodic trends for temperature, salinity, and nutrient and chlorophyll-a concentrations were also measured as representative environmental parameters. The seasonal trends in surface seawater temperature reflected the strong overall environmental seasonality of the system. Total nitrogen and phosphorus values were high and indicated that the studied waters were eutrophic [36, 37]. Against this background, the importance of gradual seasonal forcing in shaping the microbial communities in the seawater was explored. In addition, inconsistent with a previous report showing high levels of bacterial community diversity [38], our data showed that the annual patterns in the observed and predicted numbers of bacterial OTUs were similar throughout the year in Gosung Bay, and the fluctuations were not markedly cyclical, as indicated by the similar Shannon and Chao1 values among the seasonal water samples. This suggests that the bacterial taxonomic richness measured in this study was less sensitive to seasonal forcing factors. In addition, many estuaries, such as Gosung Bay, are in constant flux, with wide variations in environmental conditions, sometimes over a very short time, different seasons or different spatial scales [39, 40], leading to low levels of bacterial community diversity in oligo- and mesohaline waters of the estuary, as seen in previous studies [41, 42]. On the basis of the variability among taxa, we generated separate diversity maps for the dominant bacterial groups in order to determine whether the patterns of diversity were consistent among phyla. Diverse phylum-specific diversity patterns were found, probably reflecting the marked functional diversity encompassed by bacteria. Consistent with previous studies of the relative richness of dominant phyla [8, 38], the seasonal microbial community composition of the seawater showed some characteristics in common with oceans globally. It is well established that *Proteobacteria* and *Bacteroidetes* are the dominant bacterial phyla in seawater, and they showed different seasonal abundances. The abundance of *Proteobacteria* sequences tended to peak in summer and autumn, when light and primary production levels were high, and inorganic nutrient concentrations were at their nadir. In contrast, the *Bacteroidetes* tended to show distinct cyclical patterns in terms of their population size, with maxima in winter and minima in summer. In addition, *Cyanobacteria* were only observed during summer, whereas *Bacillariophyta* were present during autumn. The observed and predicted bacterial richness at both sampling locations exhibited cyclical seasonal change and a return of the community structure to the approximate starting point at the end of the year-long study. Although data were collected for only one year, the annual pattern was similar for both locations, which suggests that bacterial richness responded directly to seasonally fluctuating environmental parameters such as water temperature, salinity and inorganic nutrients. The conserved pattern of cyclical change implies an active seasonal response among the more dominant taxa in the microbial communities in parallel with the less numerous ones. Furthermore, in the present study, most bacteria showed temporally heterogeneous distributions over the seasons. However, their distributional patterns differ considerably from those reported previously [38, 40] and they do reverse on a seasonal basis. For example, the genus *Pelagibacter* (*Alphaproteobacteria*) is the dominant and most widely distributed bacterium in all seasons [38], but we found a pronounced peak in its relative abundance during autumn. This high relative abundance may have contributed to the low Shannon diversity value during autumn compared with those in other seasons (Table 2). Consistent with the notion that summer blooms reduce the Shannon diversity [38], *Arcobacter* was abundant during summer but was at a relatively low abundance during the other seasons. Only in the summer seawater samples was the cyanobacterial genus *Prochlorococcus* present at a relatively high abundance, which is consistent with a previous report [42]. The peaks of *Prochlorococcus* suggest the involvement of light availability in the evolution of distinct *Prochlorococcus* ecotypes [42], and its contribution to photosynthesis during summer.

CCA is powerful tool for detecting relationships between the microbiological community composition and environmental factors. Several environmental parameters, including total phosphorus, organic matter and pH, have been considered to be key factors driving changes in community composition in aquatic ecosystems. For example, CCA ordination revealed that chlorophyll-a contributed strongly to the spatial distribution of *Epsilonproteobacteria* and *Chroobacteria*, while temperature had a negative effect on the distribution of *Alphaproteobacteria*. In general, the primary product of the oceans represents an energy source available to bacteria, and chlorophyll-a may also interact to some extent with phytoplankton, which has an important influence on bacterial growth and production [43, 44]. A dramatic change in chlorophyll-a levels during summer in Gosung Bay may lead to an increase in the growth of *Pseudoalteromonas*, *Vibrio*, *Amphritea* and *Aliivibrio*, genera of the *Epsilonproteobacteria* and *Chroobacteria*. However, a large number of *Glaciecola* and *Polaribacter* were present in the cold water typifying winter samples, which is consistent with a report by *Brinkmeyer* et al. [45]. Furthermore, nitrate and dissolved oxygen also significantly influenced the bacterial community composition, including *Alphaproteobacteria*. Dissolved oxygen is particularly correlated with denitrification, and an increase in nitrate concentration can markedly promote denitrification. A higher nitrite concentration was observed in autumn samples relative to those in other samples. Differences in the mechanisms affecting oxygen concentrations among the seasons provide one explanation for the different bacterial community compositions. These results suggest that dissolved oxygen had a direct and significant effect on the bacterial communities in this study.

Interestingly, no measured environmental variable had a significant effect on the occurrence of *Gammaproteobacteria*, suggesting that its presence may be determined by other biotic and abiotic factors. In addition to physical and chemical variables, the phytoplankton composition, grazing and viral infections play important roles in shaping the structure of bacterial communities. The seasonal environmental heterogeneity in Gosung Bay may explain the differences in environmental factors that significantly determine the nature of the seasonal bacterial community. In addition, the proportion of *Vibrio* spp. in Gosung Bay increased significantly during summer compared with those in the other seasons. The effect of temperature on the abundance of *Vibrio* spp. in marine waters has been investigated [45, 46]. Elevated water temperature resulted in a notable increase in the incidence of yellow blotch disease on corals infected with *Vibrio* spp. The number of *Vibrio* spp. in the ocean is highly correlated with water temperature up to 26°C, beyond which there appears to be no additional increase in the number of bacteria [45, 46]. As the water temperature typically exceeds 26°C from May through to October in Gosung Bay, the *Vibrio* density could be expected to increase during summer, and indeed we found that species of this genus dominated samples collected at this time of year. This indicates that geographical environmental factors such as temperature are strongly associated with *Vibrio* density [47]. The susceptibility of marine organisms to disease could increase because of changes in environmental conditions that either increase the prevalence and virulence of existing pathogenic bacteria or facilitate the introduction of other pathogens, including *Vibrio* and *Arcobacter*. The proportions of *Vibrio* and *Arcobacter* were markedly higher in summer than in the other seasons. The statistical analyses suggested that distributional patterns of bacteria were driven by a combination of the seasonally changing environmental variables of temperature, salinity and inorganic nutrients.

In conclusion, in this study, changes in the bacterial diversity and structure observed over an annual cycle at two locations within Gosung Bay reflected distinct and clearly identifiable responses to seasonal environmental forcing factors. Among the environmental parameters found to be driving the variations observed in the bacterial community compositions, salinity and temperature were most influential. In particular, these factors exerted a strong influence on the *Flavobacteriaceae*, *Rhodobacteraceae*, SAR11-1 and *Vibrionaceae*. In addition, annual cycling of the environmental forcing factors appeared to reset the microbial community structure close to the same starting point at the beginning and end of our year-long study, suggesting that environmental factors result in significant and rapid reassembly of the microbial community in order to maintain biological community structure. This study has provided novel insights into the seasonal changes that structure bacterial communities in response to different environmental variables and habitats.
